# Do interoception and attending to the upper limbs affect body ownership and body representation in the grasp illusion?

**DOI:** 10.1371/journal.pone.0259988

**Published:** 2021-11-17

**Authors:** Annie A. Butler, Lucy S. Robertson, Audrey P. Wang, Simon C. Gandevia, Martin E. Héroux

**Affiliations:** 1 Neuroscience Research Australia, Randwick, NSW, Australia; 2 University of New South Wales, Randwick, NSW, Australia; 3 University of Sydney, Westmead, Australia; Anglia Ruskin University, UNITED KINGDOM

## Abstract

Passively grasping an unseen artificial finger induces ownership over this finger and an illusory coming together of one’s index fingers: a grasp illusion. Here we determine how interoceptive ability and attending to the upper limbs influence this illusion. Participants passively grasped an unseen artificial finger with their left index finger and thumb for 3 min while their right index finger, located 12 cm below, was lightly clamped. Experiment 1 (n = 30) investigated whether the strength of the grasp illusion (perceived index finger spacing and perceived ownership) is related to a person’s level of interoceptive accuracy (modified heartbeat counting task) and sensibility (*Noticing* subscale of the Multidimensional Assessment of Interoceptive Awareness). Experiment 2 (n = 30) investigated the effect of providing verbal or tactile cues to guide participants’ attention to their upper limbs. On their own, neither interoceptive accuracy and sensibility or verbal and tactile cueing had an effect on the grasp illusion. However, verbal cueing increased the strength of the grasp illusion in individuals with lower interoceptive ability. Across the observed range of interoceptive accuracy and sensibility, verbal cueing decreased perceived index spacing by 5.6 cm [1.91 to 9.38] (mean [95%CI]), and perceived ownership by ∼3 points on a 7-point Likert scale (slope -0.93 [-1.72 to -0.15]). Thus, attending to the upper limbs via verbal cues increases the strength of the grasp illusion in a way that is inversely proportional to a person’s level of interoceptive accuracy and sensibility.

## Introduction

Proprioception and the brain’s representation of the body are crucial to move autonomously and to interact with the world [1, 2]. Closely related to this is the sense of body ownership, the feeling that our body and its parts belong to us. Various illusions have been used to investigate the neurophysiological and cognitive processes that underpin body ownership, most famous of which is the rubber hand illusion [3]. In this illusion, a person’s hidden hand is brushed at the same time and in the same manner as a visible rubber hand. In many people, this induces a sense of ownership over the rubber hand and a shift in the perceived location of their real, hidden hand. Initially thought to require congruent, ongoing, multisensory stimuli [4], we devised two novel illusions —the rubber finger illusion [5] and the grasp illusion [5, 6]— to demonstrate these bodily illusions of ownership can be induced by congruent, ongoing or static stimuli that activate a single class of sensory receptors, namely cutaneous receptors or muscle spindles.

In both our illusions, the hands are hidden from view and spaced vertically 12 cm apart. One hand passively grasps an artificial finger as the other hand has its index finger grasped by two finger-like objects. In the rubber finger illusion, the hand grasping the artificial finger and the index finger being grasped are moved synchronously (or asynchronously), akin to the dynamic stimuli used in the rubber hand illusion. In the grasp illusion, there is a total lack of ongoing, dynamic stimuli. There is nothing to look at, and there is no ongoing tactile or movement stimuli to focus on. The grasp illusion is a more subtle, and thus possibly fragile, illusion, which provides a unique opportunity to investigate other factors that may influence the strength of bodily illusions of ownership.

The brain processes that determine whether or not a body part belongs to us are thought to work by Bayesian causal inference [7, 8]. They weigh up prior experiences, the current internal representation of the body, available sensory signals, the relevance, congruence and synchronicity of these sensory signals. Based on these sources of information and their weightings, the most plausible scenario, however anatomically or intellectually implausible, is selected. Given the nature of the grasp illusion, a change in the weighting of the central representation of the body may have a large impact on the strength of the illusion.

Preliminary work indicated people found it hard to focus on the static cutaneous stimuli relevant to the grasp illusion. Thus, we standardised participants’ attention by having them watch a video clip from a silent film as they passively grasp the artificial finger [6]. However, what would happen if we had them attend to the configuration and position of their upper limbs? A more veridical picture of their body should, in theory, render the illusory scenario that ‘I am holding my right index finger with my left hand’ less plausible. However, attending to the position of a hidden hand causes greater drift in its perceived location compared to attending to a visual-based computer task [9]. If this is true, having participants focus of their hidden hands may add uncertainty about their perceived location, which could see them rely more heavily on tactile cues from the thumb and index finger in contact with the artificial finger, a shift that would favour the illusory scenario that the object they are grasping is the index finger of the opposite hand [7, 10, 11]. The present study was designed to determine whether attending to the upper limbs influences the strength of the grasp illusion.

However, people differ in their ability to attend to their body [12–17]. In general terms, “Body awareness is the perception of bodily states, processes and actions that is presumed to originate from sensory proprioceptive and interoceptive afferents and that an individual has the capacity to be aware of. Body awareness includes the perception of specific physical sensations (e.g., awareness of heart activity; proprioception of limb position) as well as complex syndromes (e.g., pain; sense of relaxation; ‘somatic markers’ of emotions)” [18]. Closely related is the concept of interoception, which is the conscious perception of sensations arising from inside the body that involves aspects such as heartbeat, respiration, satiety, and the autonomic sensation relation to emotions [12, 13, 19]; although some prefer a broader definition that includes somatosensation and proprioception [20].

Initially, the potency of the rubber hand illusion appeared to correlate with *interoceptive accuracy* [21, 22]. This aspect of interoception relates to a person’s ability to detect and track internal bodily signals [23] and is typically assessed with a heartbeat counting task [24–26]. However, more recent studies have failed to replicate this finding [27–29].

Another important aspect of interoception is *interoceptive sensibility*, which relates to a person’s ability to notice and be aware of internal bodily signals [23]. It is commonly assessed with the Multidimensional Assessment of Interoceptive Awareness (MAIA-2) scale [14, 30]. To date, interoceptive sensibility has not been investigated in the context of the rubber hand illusion. Given that interoceptive accuracy and interoceptive sensibility are not strongly correlated with one another and thus likely distinct aspects of interoception [23, 31–34], we thought it important to assess both as we investigated factors that may influence the strength of the grasp illusion.

In a first experiment, we investigated whether individual differences in interoceptive accuracy and interoceptive sensibility were related to the strength of the grasp illusion. In a second experiment, we used ongoing verbal or tactile cues to investigate whether having people attend to the position and configuration of their hidden upper limbs influenced the strength of the grasp illusion. We also investigated whether these attentional effects were influenced by people’s interoceptive abilities, either accuracy or sensibility.

## Methods

### Participants

Thirty participants took part in Experiment 1 (18 males, mean age 34, SD 9.9; 28 right handed, 2 left handed) and thirty participants took part in Experiment 2 (17 males, mean age 33.7 years, SD 10.1, all right handed). Eleven participants performed both experiments. For these participants, the time between experiments was at least six months. Sample size calculations were not carried out. The number of participants was based on our previous study [6], where the first experiment closely resembled the first experiment of the present study, and the second experiment closely resembled the second experiment of the present study; in both cases we obtained accurate estimates of investigated effects.

Participants were informed about the procedures, but remained naive to the exact hypotheses tested. Also, participants were informed that their digits may come in to contact with objects, but they were given no information about the nature of these objects. All participants gave written informed consent. The experimental procedure was in accordance with the Declaration of Helsinki (2018) and approved by the University of New South Wales Human Research Ethics Committee.

### Experimental set-up

The set-up used was identical to that used by Héroux et al. [6]. In brief, the participant was seated in a stable chair at a table in an enclosed black booth. A monitor (height: 60 cm, width: 105 cm) was located approximately 70 cm in front of the participant, with the base of the monitor level with the participant’s shoulders. The midline of their body was aligned with the centre of the monitor. The participant was instructed to keep both feet flat on the floor and to not rotate their torso or head. An opaque cloth extended from the table to cover the participant’s neck and shoulders; this blocked their view of their arms and the testing apparatus. Next, the participant watched a 2-minute instructional video that outlined the experimental procedure and described the two experimental measures: perceived index finger spacing and perceived ownership (see *Experimental measures* section for details). The participant was instructed to remain relaxed throughout the experiment and to keep their eyes open.

At the start of each trial, the participant had their arms relaxed by their side. The experimenter, who could not be seen by the participant, positioned the participant’s arms. The right hand was semi-pronated with the ulnar border of the forearm resting on the table. The left arm was placed in the same posture on a support table directly above the right arm. The participant kept their hands relaxed throughout the experiment. We call this a passive grasp because there was no voluntary muscle activity and the participant’s digits and hands were resting in a grasp posture. Both index fingers were extended so they pointed to the contralateral side (right index pointed left and left index pointed right); they were separated vertically by 12 cm. The tips of the index fingers were aligned in the anterior-posterior direction and were centred on the body midline. The distal phalanx of the left and right index fingers overlapped in the horizontal plane.

**Fig 1:**
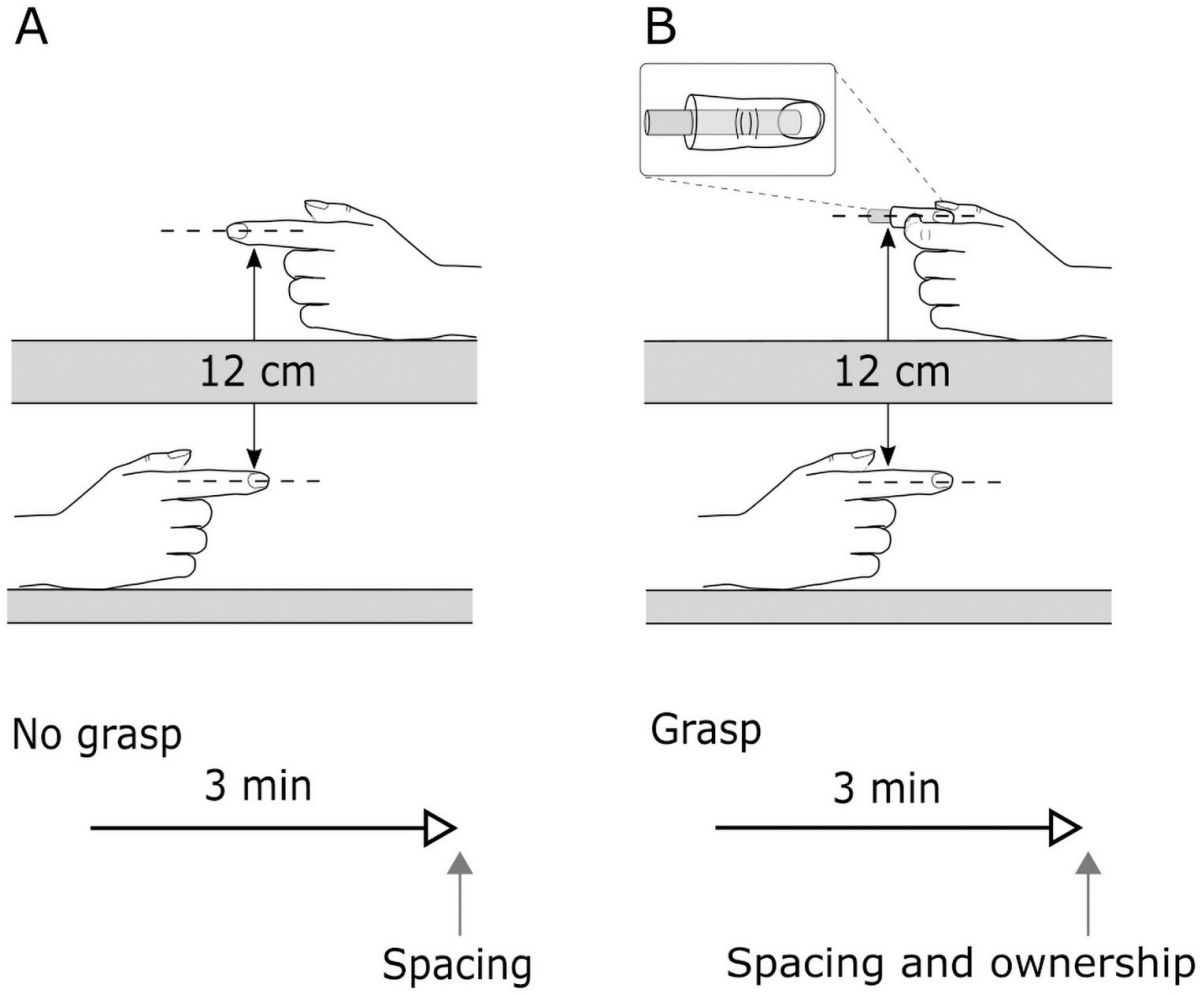
Experimental setup. There were two testing procedures, (A, Experiment 1 only) no grasp and (B) grasp. For the no-grasp condition, the right and left index fingers were separated vertically 12 cm. For the grasp condition, the hands were placed in the same position but the participant passively grasped an artificial finger with their left index finger and thumb, while their right index finger was grasped by a silicone covered clamp (not depicted for clarity). The testing apparatus and participant’s arms and hands were hidden from view.

For the no-grasp condition, the participant’s hands were positioned as described above. In the grasp condition, the participant’s hands were also positioned as described above, but the tip of their right index finger was held in position by a clamp that applied light pressure to the anterior and posterior aspect of the distal phalanx (see Fig 1). The experimenter also lightly clamped the participant’s left thumb and index finger around an artificial finger as to passively grasp it. The inner surface of both clamps was fitted with silicone, moulded in the shape of a finger. The clamps were adjusted so that the participant reported equal pressure on their left index finger and thumb, as well as on their right index finger. The artificial finger was vertically aligned 12 cm above the right index finger. It was made from silicone and had a central metal bar (diameter 0.8 cm, length 3 cm) to mimic a bone. The duration of each experimental session was approximately 15 min.

### Experimental measures

As in our previous studies [5, 6], two measures quantified the grasp illusion: perceived vertical spacing between the participant’s left and right index fingers (termed ‘perceived index finger spacing’) and perceived ownership of the artificial finger. All measures were taken at the end of the trials with the participant’s upper limbs still in position.

#### Perceived index finger spacing

In response to the question “Which line corresponds to the vertical distance between the tips of your index fingers (left or right above)”, the participant was asked to select from a series of 21 vertical lines presented on the monitor. The vertical lines were labelled with letters and ranged in height from 0 cm to 20 cm, in 1 cm increments. The lines were presented in a random order for each trial. When perceived index finger spacing was different from 0 cm, the participant indicated if they felt their left or right index finger was above.

#### Perceived ownership

For the grasp condition, the participant was asked about their level of perceived ownership over the passively grasped artificial finger. Specifically, the participant indicated how strongly they agreed or disagreed with the following statement: “I feel that I am holding my right finger index with my left hand”. The participant rated their level of agreement by selecting from a seven-point Likert scale (1–7): strongly disagree, disagree, somewhat disagree, neither agree nor disagree, somewhat agree, agree, and strongly agree.

#### Interoceptive accuracy

Interoceptive accuracy was measured with the heartbeat counting task using a modified version of the mental tracking method [25]. Heart rate was recorded with a PulseSensor (World Famous Electronics, IIc.) attached to the participant’s non-dominant index finger connected to an Arduino Uno microcontroller. To ensure the participant could not feel their arterial pulse (confirmed by the participant), the cuff of the PulseSensor was placed loosely around the finger. Additionally, the participant was instructed to not cross their legs to avoid pulse-related cues. The participant was instructed to silently count the number of heartbeats they felt without physically measuring their heartbeat. The experimenter gave a “start” cue and 40 seconds later a “stop” cue. The participant was then asked to indicate the number of heartbeats they counted. The participant was not aware of the duration of the trial. Importantly, the participant was instructed to only report the number of heartbeats felt, thus zero was a possible answer. Emphasising that counted heartbeats must be felt minimised the chance that the participant guessed an answer based on knowledge of their resting heartrate and an estimate of the duration of the trial; these modified instructions improve the validity of the heartbeat counting task [35–37]. The participant was not given any feedback in their performance in the task. Performance on this task was measured as:

$$\begin{array}{c} \text{Heartbeat}\;\text{counting}\;\text{task}\;\text{score}=1-\frac{\left(|actual\;heartbeats-reported\;heartbeats|\right)}{actual\;heartbeats} \end{array}$$

Thus, possible values ranged from 0 to 1, where 0 represents not feeling a single heartbeat (low interoceptive accuracy) and 1 represents feeling every heartbeat (high interoceptive accuracy).

#### Interoceptive sensibility

Interoceptive sensibility was measured with the *Noticing* subscale of the MAIA-2 scale [14, 30]. It assesses a person’s awareness of uncomfortable, comfortable, and neutral body sensations, and forms the *Awareness of Body Sensations dimension* of the MAIA-2 scale. The *Noticing* subscale includes four questions scored on a 0 to 5 ordinal scale, with 0 associated with the anchor word ‘never’ and 5 associated with the anchor word ‘always’. The four questions are: ‘When I am tense I notice where the tension is located in my body’, ‘I notice when I am uncomfortable in my body’, ‘I notice where in my body I am comfortable’, and ‘I notice changes in my breathing, such as whether it slows down or speeds up’. The average score across the four questions was computed. Thus, possible values ranged from 0 to 5, with higher average scores reflecting greater interoceptive sensibility.

### Experimental protocol

#### Experiment 1

This experiment assessed whether individual differences in interoceptive accuracy and interoceptive sensibility were related to the strength of the grasp illusion. It consisted of two conditions: no-grasp and grasp (see Fig 1). Each condition was tested on a separate day, with at least two days between testing sessions. The order of conditions was randomised across participants. Each trial began with the experimenter positioning the participant’s hands. Next, the participant viewed a 3 min video clip from a silent film, which standardised attention across participants and trials. The video clip on the second day was the continuation of the same silent film. Immediately following the video clip, perceived index finger spacing (no-grasp and grasp conditions) and perceived ownership (grasp condition only) were measured. Interoceptive accuracy (heartbeat counting task) and interoceptive sensibility (*Noticing* subscale) were measured at the end of the testing sessions. The order of these measures was randomised across participants.

#### Experiment 2

This experiment assessed whether ongoing verbal or tactile cues designed to have participants attend to the position and configuration of their upper limbs influenced the strength of the grasp illusion. It also assessed whether these attentional effects are influenced by a person’s interoceptive accuracy or sensibility. This experiment consisted of three variations of the grasp condition from Experiment 1: control (video), verbal cueing, and tactile cueing. Each condition was tested on a separate day, with at least two days between testing sessions. The order of testing was randomised across participants. Interoceptive ability was measured at the end of the second and third testing session, with the heartbeat counting task on one day and the *Noticing* subscale on the other. The order of these measures was randomised across participants.

**Control (video)**. This condition was identical to the grasp condition described in Experiment 1, except that the participant was told they would be quizzed on the content of the 3-min video clip. Immediately following the video clip, perceived index finger spacing and perceived ownership were measured, followed by three questions about the video.

**Verbal cues**. This condition examined how verbal instructions designed to have the participant focus on their upper limbs influenced perceived index finger spacing and perceived ownership. Rather than watch a silent video clip, the participant wore headphones and listened to a 3-min body scan. The body scan asked the participant to focus on the location of, and sensations arising from, their shoulders, arms, elbows, forearms and hands. Approximately 10–15 s were spent on each body part, with the full upper limb body scan repeated twice over the 3-min recording. The participant was instructed to focus on the body part in time with the verbal instructions. Immediately after this, perceived index finger spacing and perceived ownership were measured.

**Tactile cues**. This condition examined how tactile stimuli designed to have the participant focus on their upper limbs influenced perceived index finger spacing and perceived ownership. Rather than watching a silent video clip, the participant focused on the tactile stimuli applied to their shoulders, arms, elbows, forearms and hands. The tactile stimuli were applied with a smooth rounded wooden stick (8 mm diameter) moved over the skin at approximately 5 cm/s. The tactile cueing of this condition was paired with the verbal cueing of the previous condition. To accomplish this, the experimenter listened to the recorded body scan through headphones and applied the tactile cueing in sync with the verbal instructions. For example, if the verbal body scan asked the participant to focus on their left forearm, the experimenter, hearing the body scan through the headphones, stroked the participant’s left forearm. The participant was instructed to focus on the tactile stimuli for the duration of the 3-min trial. Halfway through the trial, participants were verbally reminded to attend to the tactile stimuli. At the end of the trial, perceived index finger spacing and perceived ownership were measured.

### Statistical analysis

All analyses were pre-planned and no additional exploratory analyses were conducted. Data were analysed with an estimation approach based on confidence intervals [38–40]. In Experiment 1 we quantified the effect of the grasp illusion on perceived index finger spacing and perceived ownership (grasp condition only). For perceived index finger spacing, the mean [95% CI] difference between the two conditions (grasp–no grasp) was calculated. For perceived ownership, items from the Likert scale were paired to an integer scale ranging from 1 (strongly disagree) to 7 (strongly agree) and the mean [95% CI] was calculated. Ordinary least-squares multiple regression was used to determine whether interoceptive measures (heartbeat counting task, *Noticing* subscale) were associated with the strength of the grasp illusion (grasp–no grasp difference in perceived index finger spacing, perceived ownership).

Except for the addition of the interoceptive measures, Experiment 1 was identical to Experiment 1 of our previous study [6]. Thus, to obtain more precise estimates of the effects associated with the grasp illusion, perceived index finger spacing and perceived ownership results from both studies were pooled. This is equivalent to performing a two-study meta-analysis.

In Experiment 2, we quantified the effect of verbal and tactile cueing related to the position and configuration of the upper limb on the grasp illusion by comparing results from the control (video) condition to those of the two cueing conditions. Specifically, we calculated mean [95% CI] differences (control–verbal; control–tactile) for perceived index finger spacing and perceived ownership. Ordinary least-squares multiple regression was used to determine whether the interoceptive measures (heartbeat counting task, *Noticing* subscale) were correlated with the effects of verbal or tactile cueing on the grasp illusion.

In Experiment 1, Pearson’s correlation was used to measure the association between the two measures of the grasp illusion (perceived index finger spacing [grasp–no grasp] *versus* perceived ownership over the artificial finger). In both experiments, Pearson’s correlations were also used to measure the association between the measure of interoceptive accuracy (heartbeat counting task) and interoceptive sensibility (*Noticing* subscale).

All primary values and all primary difference values are included in figures. All summary values reported in text and figures are means and 95% CI, except for the mean ± standard deviation which is reported for the age of participants. All data, code and generated outputs are publicly available (S1 File).

## Results

### Experiment 1

**Fig 2:**
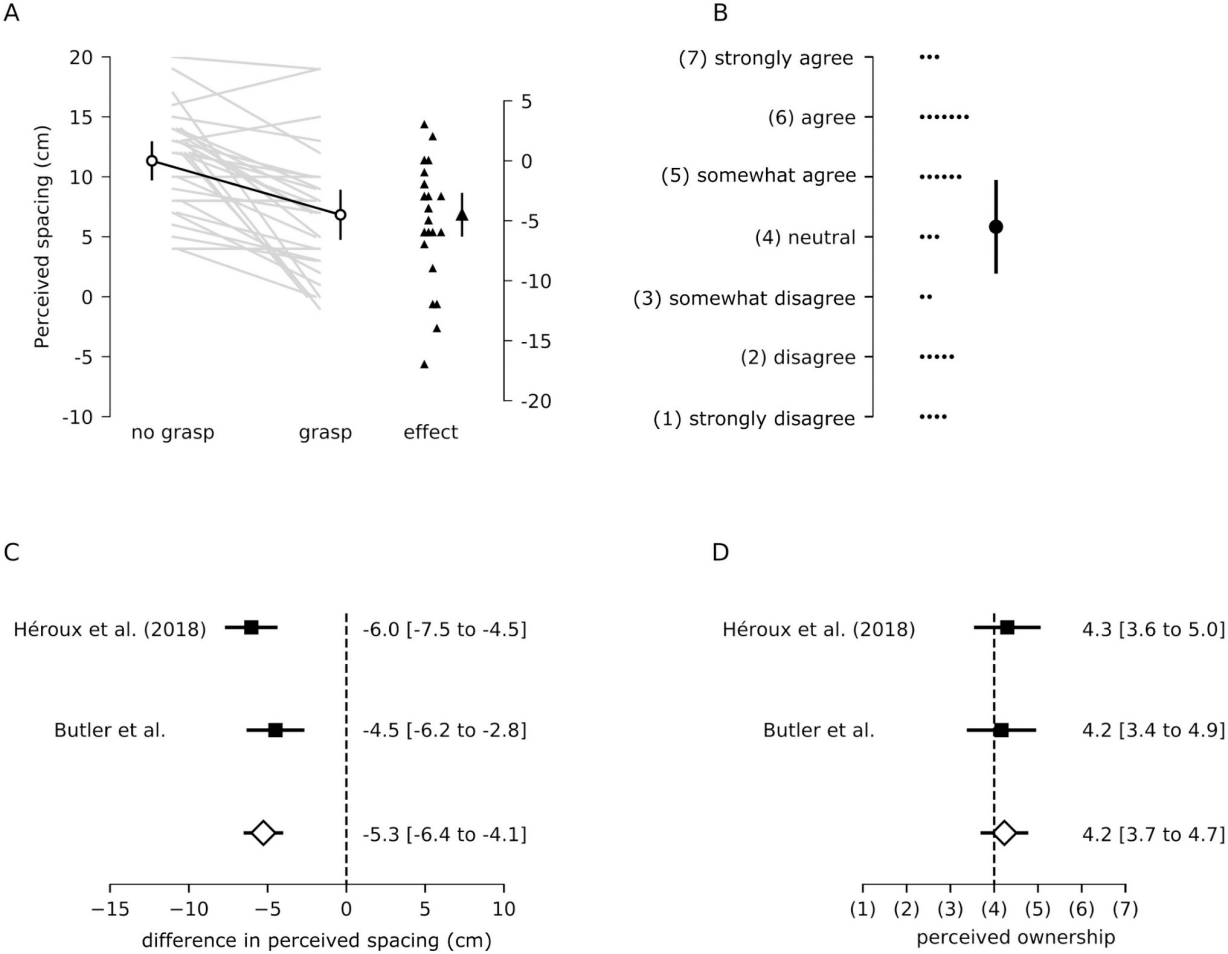
Experiment 1 results and meta-analysis. (A) Perceived index finger spacing for the no-grasp and grasp conditions for each participant (grey lines). The difference in perceived index finger spacing is also plotted for each participant (black triangles; effect: grasp–no grasp). (B) Perceived ownership for each participant. Also plotted are the pooled results of the current study (Butler et al., n = 30) and our previous study (Héroux et al. [6], n = 30) for (C) the difference in perceived spacing (grasp–no grasp) and (D) perceived ownership. The pooled effect is depicted by the open diamonds. All values are means [95% CI].

This experiment examined how individual differences in interoceptive accuracy and sensibility were related to the strength of the grasp illusion. Perceived index finger spacing was, on average, 11.3 cm [9.8 to 12.9] (mean [95%CI]) for the no-grasp condition and 6.8 cm [4.8 to 8.8] for the grasp condition. Thus, participants perceived their index fingers to be 4.5 cm [2.8 to 6.2] closer together when grasping an artificial finger (Fig 2A). Furthermore, perceived ownership was, on average, 4.2 [3.4 to 4.9] for the grasp condition (Fig 2B), slightly above ‘neither agree or disagree’ on the Likert scale. These results replicate those of our previous study [6]. The pooled estimates indicate that passively grasping an artificial finger for 3 min reduces perceived index finger spacing by 5.3 cm [4.1 to 6.4] (Fig 2C) and is associated with a sense of perceived ownership of 4.2 [3.7 to 4.7], between ‘neither agree or disagree’ and ‘somewhat agree’ (Fig 2D).

In line with previous results [5], perceived ownership over the artificial finger was negatively correlated with perceived index finger spacing (r = -0.44, [95%CI -0.69 to -0.10]: greater perceived ownership was associated with a greater coming together of the hands.

The average score for the *Noticing* subscale was 3.2 [2.9 to 3.6] and the average score on the heartbeat counting task was 0.67 [0.57 to 0.78]. Values from these two measures were not correlated (r = 0.19, [95% CI -0.18 to 0.51]). Multiple regression analysis revealed that interoceptive accuracy and interoceptive sensibility were not related to the strength of the grasp illusion (Table 1).

**Table 1:** Associations between interoceptive measures and the strength of the grasp illusion.

	R^2^	Coefficient	SE	*t*	*P*	95% CI
**Grasp illusion—perceived spacing**	0.03					
Intercept		7.31	3.79	1.93	0.065	-0.47 to 15.09
Interoceptive sensibility –*Noticing* subscale		-0.31	1.07	-0.29	0.777	-2.49 to 1.88
Interoceptive accuracy—Heartbeat counting		-2.70	3.17	-0.85	0.401	-9.20 to 3.79
**Grasp illusion—perceived ownership**	0.01					
Intercept		3.26	1.67	1.95	0.061	-0.17 to 6.69
Interoceptive sensibility –*Noticing* subscale		0.24	0.47	0.50	0.619	-0.73 to 1.20
Interoceptive accuracy—Heartbeat counting		0.21	1.39	0.15	0.881	-2.65 to 3.07

### Experiment 2

**Fig 3:**
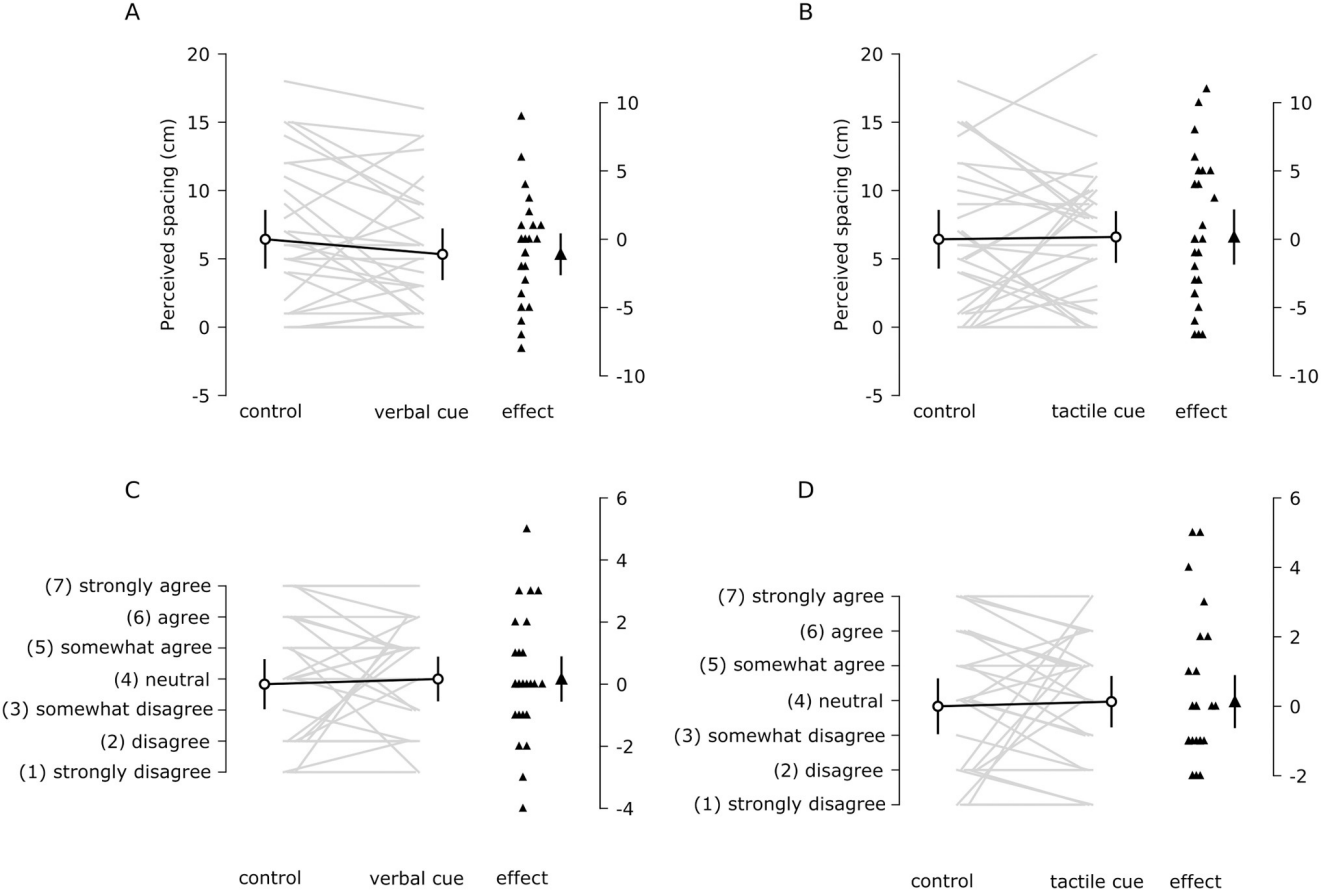
Experiment 2 results. Perceived index finger spacing in the control (video) condition and the (A) verbal and (B) tactile cueing conditions for each participant (grey lines). The difference in perceived index finger spacing is also plotted for each participant. Perceived ownership during the control (video) condition and the (C) verbal and (D) tactile cueing conditions for each participant. The difference in perceived ownership for each participant is also plotted (verbal–control). All values are means [95% CI].

This experiment examined whether directing a person’s attention to their upper limbs with verbal or tactile cues affects the strength of the grasp illusion. Compared to attending to a 3-min silent video clip (control condition), 3 min of verbal cueing had little to no effect on perceived index finger spacing (-1.1 cm [-2.6 to 0.4], (mean [95%CI]), Fig 3A or perceived ownership (0.2 [-0.5 to 0.9], Fig 3C). Similarly, 3 min of tactile cueing had little to no effect on perceived index finger spacing (0.2 cm [-1.8 to 2.1], Fig 3B) or perceived ownership (0.1 [-0.6 to 0.9], Fig 3D).

**Fig 4:**
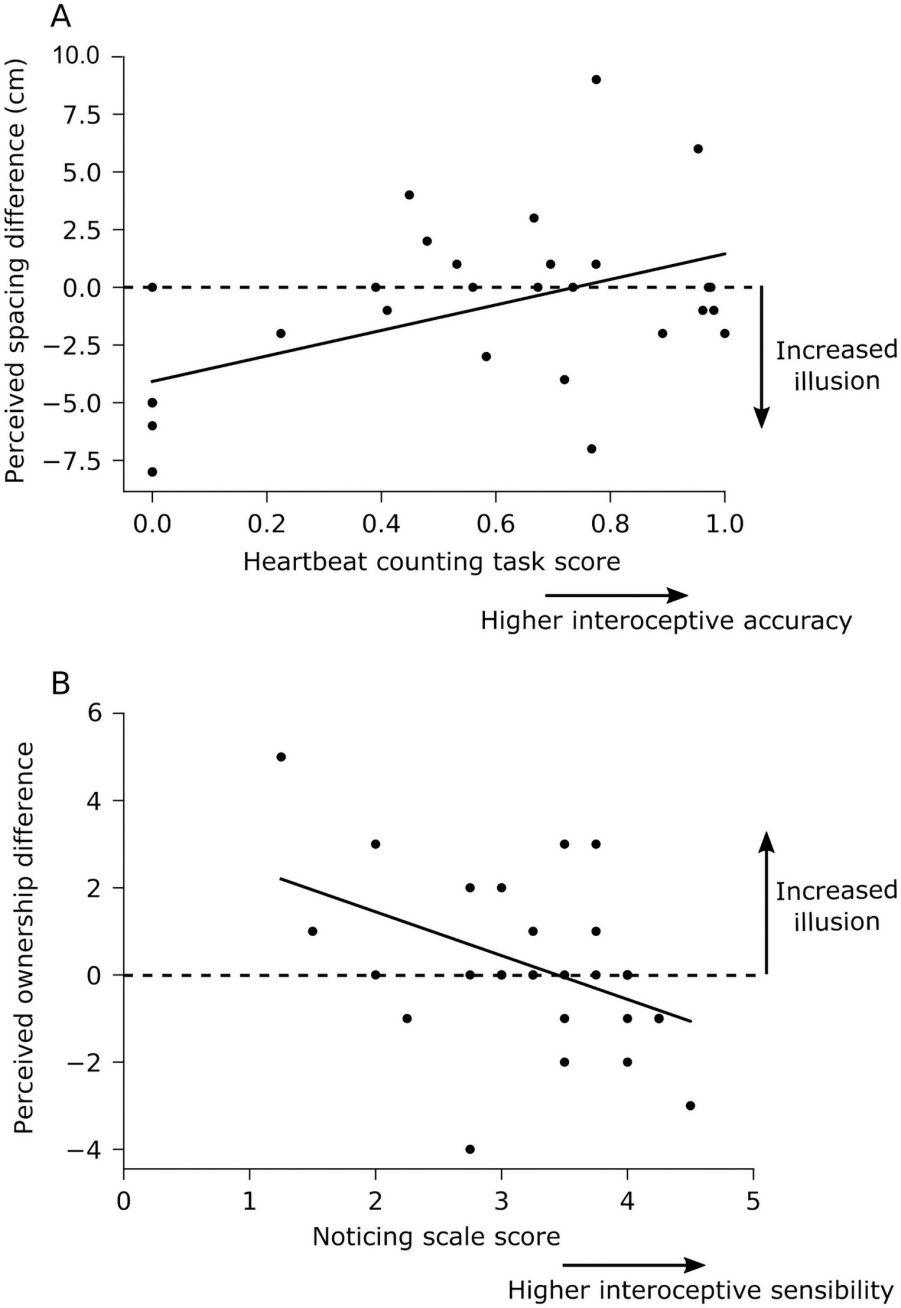
Relationship between measures of interoception and the difference in grasp illusion between the control (video) condition and the verbal cueing condition. (A) Multiple regression analysis revealed a relationship between performance on the heartbeat counting task and the difference in perceived index finger spacing between the control (video) and verbal cueing conditions (regression coefficient [95%CI]: 5.64 [1.91 to 9.38]). This indicates that, compared to the control (video) condition, verbal cueing was associated with the hands feeling closer together in participants with worse interceptive accuracy. (B) Multiple regression analysis revealed a relationship between average score from the *Noticing* subscale and the difference in perceived ownership between the control (video) and verbal cueing conditions (regression coefficient: -0.93 [-1.72 to -0.15]). This indicates that, compared to the control (video) condition, verbal cueing was associated with a stronger illusion of owning the artificial finger in participants with worse interoceptive sensibility. The dotted line represents no difference between the control (video) and verbal cueing conditions.

However, regression analysis revealed that interoceptive accuracy was related to the effect verbal cues had on the strength of the grasp illusion (Table 2, Fig 4A).

**Table 2:** Associations between measures of interoception and the effects of attention on the grasp illusion.

	R^2^	Coefficient	SE	*t*	*P*	95% CI
**Control vs verbal—perceived spacing**	0.26					
Intercept		-3.19	2.67	-1.19	0.243	-8.67 to 2.29
Interoceptive sensibility –*Noticing* subscale		-0.29	0.79	-0.37	0.714	-1.91 to 1.32
Interoceptive accuracy—Heartbeat counting		5.64	1.82	3.10	0.005	1.91 to 9.38
**Control vs verbal—perceived ownership**	0.23					
Intercept		3.73	1.30	2.88	0.008	1.07 to 6.40
Interoceptive sensibility –*Noticing* subscale		-0.93	0.38	-2.44	0.021	-1.72 to -0.15
Interoceptive accuracy—Heartbeat counting		-0.94	0.89	-1.06	0.299	-2.76 to 0.88
**Control vs tactile—perceived spacing**	0.01					
Intercept		0.07	4.12	0.02	0.987	-8.39 to 8.52
Interoceptive sensibility –*Noticing* subscale		-0.22	1.21	-0.18	0.858	-2.71 to 2.72
Interoceptive accuracy—Heartbeat counting		1.51	2.81	0.54	0.595	-4.25 to 7.28
**Control vs verbal—perceived ownership**	0.04					
Intercept		1.14	1.53	0.74	0.464	-2.01 to 4.29
Interoceptive sensibility –*Noticing* subscale		-0.42	0.45	-0.92	0.366	-1.34 to 0.51
Interoceptive accuracy—Heartbeat counting		0.66	1.05	0.63	0.533	-1.49 to 2.81

For perceived index finger spacing, performance in the heartbeat counting task was associated with a model coefficient of 5.64 [1.91 to 9.38]. This indicates that, if we compare a person with poor interoceptive accuracy who scored 0 on the heartbeat counting task to a person with good interoceptive accuracy who scored 1 on this task, verbal cueing would be associated with an average difference in perceived index finger spacing of 5.6 cm between these two people. Fig 4A shows each participant’s heartbeat counting score plotted against their difference in perceived index finger spacing between the verbal cueing condition and the control (video) condition.

For perceived ownership, scores from the *Noticing* subscale were associated with a model coefficient of -0.93 [-1.72 to -0.15]. This indicates that, for each 1-point increase in a participant’s average *Noticing* subscale score, the difference in perceived ownership between the verbal cueing condition and the control (video) condition decreases by, on average, 0.93 on a 7-point Likert scale. Fig 4B shows each participant’s average *Noticing* subscale score plotted against their difference in perceived ownership between the verbal cueing condition and the control (video) condition.

Similar to the results of Experiment 1, there was no association between the measures of interoceptive accuracy and sensibility (r = 0.17, [95% CI -0.20 to 0.50]).

## Discussion

Our results indicate that the strength of the grasp illusion is not directly influenced by a person’s interoceptive ability, whether it be interoceptive accuracy or interoceptive sensibility. Moreover, verbal and tactile cues, in and of themselves, do not affect the strength of this illusion. However, verbal cueing does increase the strength of the grasp illusion in people with lower interoceptive accuracy and interoceptive sensibility. Thus, attending to the position and configuration of ones upper limbs influences the strength of a bodily illusion of ownership in a way that depends on a person’s interoceptive ability. The size of this interaction is substantial and underscores the complexity of the physiological and cognitive processes that govern the human sense of body ownership.

### Experiment 1

This experiment replicated our initial results [6], and pooling data from both studies generated more precise estimates of the grasp illusion. We also replicated the moderate relationship between perceived index finger spacing and perceived ownership observed in our previous study: perceiving one’s index fingers closer together is correlated with stronger ownership of the grasped artificial finger.

Importantly, the strength of the grasp illusion was not related to a person’s level of interoceptive accuracy, which is in line with some [27–29], but not all previous studies [21, 22]. Moreover, it was not related to scores from the *Noticing* subscale the MAIA-2 scale, which capture the Awareness of Bodily Sensations dimension of interoceptive sensibility. Based on the available evidence, we believe that, in isolation, interoceptive abilities have little effect on the strength of illusions of body ownership.

In the present study we focused on a single dimension of interoceptive sensibility. However, others were possible. The subscales we considered but, in the end, deemed less relevant were the *Attention Regulation* and *Body Listening* subscales. The *Attention Regulation* subscale, which is correlated with the *Noticing* subscale (r = 0.56) [14], assesses a person’s ability to stay focused when faced with numerous sensory stimuli competing for attention, whereas the *Body Listening* subscale assesses whether a person ‘Actively listens to their body for insight’, with two of its three statements asking about a connection between body sensations and emotions.

We also focused on a single ownership statement, an approach previously adopted by us and others [4–6, 41]. A single statement allows perceived ownership to be assessed quickly, as participants are still experiencing the grasp illusion. The original Botvinick and Cohen [3] questionnaire included nine questions, which are typically administered after the rubber hand illusion protocol is complete, sometimes after participants have removed their hands from the experimental apparatus. Although it was never the intention, some groups include one or more of these nine questions to control for, or assess, participant compliance and suggestibility [42–44].

The experiential effects related to the rubber hand illusion can be ordered with respect to how likely they are to be reported [45]. This ordering highlights two important points. First, these effects are similar across people; they follow the same continuum. Second, a person’s susceptibility to the rubber hand illusion is what determines the number of questions they will respond positively to. Thus, positive responses to questions 4 to 9 from the original rubber hand illusion study [3] likely reflect a person’s susceptibility to this illusion, not their susceptibility in general.

In future, additional MAIA-2 subscales could be assessed to investigate other links between interceptive sensibility and illusions of body ownership. Similarly, it may be relevant to include additional ownership questions, as this would capture people’s susceptibility to these illusions, not just their presence or absence.

### Experiment 2

Compared to the control video condition, verbal or tactile cueing did not, by themselves, alter the overall strength of the grasp illusion. However, the effect of cueing varied greatly across participants: what might account for this?

Part of the answer appears to lie in individual differences in interoceptive accuracy and interoceptive sensibility. In the present study, the more objective measure of interoception, heartbeat counting, was related to the more objective measure of the grasp illusion, perceived index finger spacing, while the more subjective measure of interoception, the *Noticing* subscale, was related to the more subjective measure of the grasp illusion, perceived ownership. In line with previous studies [23, 31, 32], these two measures of interoceptive ability were not correlated with one another. This supports the view that interoceptive accuracy and sensibility represent relatively distinct facets of interoception. Furthermore, in both cases, lower interoceptive ability was associated with a stronger grasp illusion. That is, verbal cueing increased the strength of the grasp illusion in people less in tune with their bodily sensations.

In our sample of 30 participants, the lowest average score on the *Noticing* subscale was 1.2. We did not have participants with the lowest level of interoceptive sensibility possible (i.e., average score of 0). However, if the relationship noted in the current study holds across the entire range of potential scores, these people would experience an average increase of ∼4 points in perceived ownership with verbal cueing. Conversely, if we extrapolated our results to people with the highest level of interoceptive sensibility, verbal cueing would actually decrease perceived ownership by ∼1 point. Although interesting, this remains speculative. Future experiments should include participants with extremely high and extremely low interoceptive sensibility.

Why would being told to focus attention on the position and configuration of the hidden upper limbs lead to stronger grasp illusions in people with low interoceptive ability? It may be that, in people with low interoception, actively focusing on their upper limbs highlights just how uncertain they are about the position and configuration of their upper limbs. Thus, these individuals may give more importance to, or weight more heavily, tactile inputs directly related to the illusion. Conversely, in people with high interoceptive ability, actively focusing on the position and configuration of their upper limbs may bring additional certainty of their location in space, which would reduce the likelihood that the object being grasped is their own index finger.

Also important is the precision with which we estimated the interaction between interoceptive ability and verbal cueing on the grasp illusion. If we consider perceived index finger spacing, the model coefficient for heartbeat counting scores during verbal cueing was 5.64 [1.92 to 9.38]. Thus, the true population effect could be as small as 1.92 or as large as 9.38. This is a lot of uncertainty, an observation that also applies to the relationship between average scores on the *Noticing* subscale and the effect of verbal cueing on perceived ownership (-0.93 [-1.72 to -0.15]). While the precision of our estimates would be improved with a larger sample size, the uncertainty associated with these estimates may suggest that other factors contribute to, or mediate, the effect of verbal cueing on the strength of the grasp illusion.

One such factor is the inclusion of the digits involved in the grasp illusion into the body scan. This could strengthen or weaken the illusion. However, our preliminary work found that people had difficulty focusing on the static, cutaneous stimuli involved in the grasp illusion. Thus, if future studies investigate this factor, it would be important to assess, or control for, participant compliance with the task, something that is not yet commonplace in most studies of bodily illusions of ownership.

Another factor to consider is proprioceptive accuracy. A recent study found a positive relationship (r = 0.30 to 0.33) between proprioceptive accuracy, as measured by a joint position matching task, and the strength of the rubber hand illusion [28]. Superficially, it is not unreasonable to think that a person with high proprioceptive accuracy has a better sense of where their limbs are located in space. However, matching tasks do not assess the aspect of position sense that allows us to determine where our limbs are located in space [46–48]. Thus, it would be informative to replicate these findings and determine whether they differ when a more relevant aspect of position sense is assessed.

In future, if these and other relevant factors are considered in the same study, causal mediation analysis could be used to determine which factors drive a person’s susceptibility to illusions of body ownership [49].

It is not entirely clear why tactile cueing did not have the same effect as verbal cueing. In this experimental condition, participants were instructed to focus on the tactile stimuli. Thus, their attention was likely consumed by the focal stimuli, not the location or configuration of their upper limbs. This difference in what is being attended to may explain the limited effect tactile cueing had on the grasp illusion. It may be that participants focused on the tactile stimuli and where it was happening on the body, without determining where it was happening in the external world. In future, if we repeat this study, we plan to have participants focus on the tactile stimuli applied to their upper limbs *and* where in space it was taking place, as this may be a crucial distinction.

We used a heartbeat counting task to assess interoceptive accuracy [21, 25, 50]. Specifically, we used the modified instructions for the mental tracking method [25, 35, 36] as this avoid participants getting high scores based on knowing their resting heart rate and estimating the duration of the trial [50, 51]. However, given the task was assessed only once, our measure of interoceptive accuracy is susceptible to random measurement error.

## Conclusion

Verbal cues that bring attention to the upper limbs are linked with stronger body ownership illusions in people with low interoceptive ability. In future, studies of body ownership should consider controlling participants’ attention and including measures of interoceptive ability—both accuracy and sensibility—as experimental factors or covariates in their statistical analyses. If our findings generalise to patient populations, a person’s interoceptive ability should be considered when optimising the therapeutic effect of body ownership illusions.

## Supporting information

S1 File Data, code and results. Directory structure with all data, code and generated outputs for the present study. Please refer to the README.md file for details.
(ZIP)
